# The effect of benzylpenicillin prohylaxis after birth on length and weight of syphilis-exposed infants in eastern China

**DOI:** 10.1186/s13052-024-01779-7

**Published:** 2024-09-27

**Authors:** Kejin Chen, Xiaoyan Zhao, Wei Wu, Lihua Jiang, Xiaojie Yuan, Chaorong Bian

**Affiliations:** 1 Changzhou Maternal and Child Health Care Hospital, Changzhou Medical Center, Nanjing Medical University, Changzhou, 213000 Jiangsu China; 2 Medical Innovation Research Department, Chinese People’s Liberation Army General Hospital, Beijing, 100000 China; 3 Affiliated Changzhou Children’s Hospital of Nantong University, Changzhou, 213018 Jiangsu China

**Keywords:** Syphilis, Infant, Body height, Body Weight, Penicillin G Benzathine, East Asian people

## Abstract

**Background:**

We conducted this study to assess the impact of an intervention to interrupt mother-to-child transmission on the height and weight of syphilis-exposed infants after receiving penicillin prophylaxis after birth and to provide a scientific basis for further elimination of mother-to-child transmission.

**Methods:**

We recruited 419 infants born to syphilis-infected mothers from 2015 to 2020 in Changzhou, and performed 1:1 matching to infants born to syphilis-free mothers during the same period. All infants were followed up to 18 months of age. We collected height and weight data and compared them.

**Results:**

At 18 months of age, the height and weight of the syphilis-exposed infants were almost greater than the WHO reference standards. However, when compared with local unexposed infants, there were almost no differences. The boys born to mothers who received two courses of treatment had longer body lengths at 18 months of age than did those born to mothers who did not receive two courses of treatment, and the girls born to mothers who did not receive treatment had lower body weights at 3 months of age than did both treated groups.

**Conclusion:**

The growth trajectory of infants without congenital syphilis born to syphilis-infected mothers is virtually indistinguishable from that of the general local population. Syphilis-exposed newborns can receive preventive treatment as a public health intervention.

**Supplementary Information:**

The online version contains supplementary material available at 10.1186/s13052-024-01779-7.

## Background

According to World Health Organization estimates, more than 6 million people between the ages of 15 and 49 years are infected with syphilis every year worldwide. In addition, the disease causes more than 300,000 foetal and neonatal deaths [1]. A study released in 2013 indicated that the total incidence of maternal syphilis in China was estimated to be 0.30% (95% CI: 0.28–0.32) [2]. Due to China’s large population, the number of syphilis-infected mothers in China is staggering [3]. Adequate resources and intervention strategies should target not only perinatal emergencies but also the management of mild pathologies, including maternal syphilis. Due to effective intervention strategies and the low cost of penicillin in recent years, pregnancies are rarely complicated by syphilis in China.

Sexually transmitted infections ranked 10th among the top ten causes of disability adjusted life years (DALYs) in children younger than 10 years in 2019, with congenital syphilis accounting for majority of cases in this age group [4]. It is gratifying to note that by the end of 2020, the national annual reported incidence of congenital syphilis was less than 15/100,000 live births in China, with the goal of eliminating congenital syphilis almost being achieved. However, it seems that a larger group may have been overlooked: syphilis-exposed children.

Previous studies on children exposed to infectious diseases have tended to focus on the incidence, influencing factors, and prevention [5, 6], and studies on child growth and development have tended to focus on some major infectious diseases, such as HIV [7]. Previously, regarding the growth and development of children with syphilis, only the group of children with congenital syphilis has received attention [8], and less attention has been given to groups of children exposed to syphilis or with insufficient numbers of affected children [9, 10]. Although China’s National Health Commission requires primary care units to follow up on infants born to syphilis-infected mothers, there is still a lack of height and weight comparisons of syphilis-exposed children with healthy children of the same age.

Previous studies by our team have shown an association between maternal syphilis treatment and serological transfer in syphilis-exposed children; [11]however, our team has not explored the relationship between maternal syphilis treatment and child growth and development. Although a higher risk of preterm birth and small for gestational age (SGA) was observed among mothers who had syphilis (OR: 12.800, 95% CI: 1.250-131.041) [12], there is insufficient evidence to conclude that the growth and development of syphilis-exposed children are affected.

Therefore, our team conducted a scientific study of this long-term preventive public health intervention in China to evaluate the effects on morphological indicators of treated syphilis-exposed infants. Relying on the China Prevention of Mother-to-child Transmission of Syphilis Information System, we followed up syphilis-exposed infants born in Changzhou from 2015 to 2020 for at least 18 months and conducted a comparative analysis of the height and weight of these infants with healthy infants, aiming to facilitate China’s early achievement of the prevention of mother-to-child transmission (PMTCT).

## Methods

### Study design and participants

This was a retrospective cohort study from Changzhou, a representative city in Jiangsu Province, China. Changzhou is a city in the center of the Yangtze River Delta, the fifth city with trillion-dollar Gross Domestic Product (GDP) in Jiangsu Province, the third in Jiangsu and the ninth in China in terms of per capita GDP. Syphilis-exposed infants born from 2015 to 2020 were included in the study, and they were defined as infants delivered by mothers diagnosed with syphilis infection during pregnancy. We excluded women with a history of miscarriage, stillbirth, or multiple births. In this study, syphilis-infected mothers had a positive rapid plasma reaction (RPR) test and a positive Treponema pallidum particle agglutination (TPPA) test during pregnancy.

We recruited a total of 419 syphilis-exposed infants (222 boys and 197 girls) born to mothers with syphilis. Based on delivery time, we randomly matched (1:1) mothers with syphilis with mothers without syphilis in the Jiangsu Maternal and Child Information System for the same period. The survey did not focus on head circumference, although neurosyphilis may be among the most serious consequences of the infection. Other congenital diseases or defects that lead to overgrowth or conversely to impaired growth have been excluded [13–15]. 

### Treatment and follow-up

According to the treatment protocol issued by the Chinese National Health Care Commission in 2015, the standard treatment for pregnant women infected with syphilis must meet the following three conditions simultaneously: [16]adequate penicillin treatment; two courses of treatment during pregnancy, with an interval of more than 2 weeks; and a second course of treatment administered and completed in late pregnancy. The prophylactic regimen for newborns is a split injection of benzylpenicillin G into both buttock muscles immediately after birth. The treatment regimen for pregnant women with syphilis infection is benzylpenicillin, 2.4 million units, intramuscularly in both gluteal muscles, once a week for 3 consecutive sessions. The regimen for prophylactic treatment of their newborns is benzylpenicillin, 50,000 units/kg body weight, 1 intramuscular injection (into both gluteal muscles).

According to the Guidelines for the Diagnosis and Treatment of Syphilis, Gonorrhea, and Chlamydia Trachomatis Infections of the Reproductive Tract (2020) issued by the Center for STD Control of the Chinese Center for Disease Control and Prevention (CDC) [17], the newborns evaluated in this study were not considered to be infected with syphilis. Even though some of them had positive serological titers after birth, they were not recognized as congenital syphilis infections because they did not exceed four times their mothers’ pre-delivery titers.

Follow-up of syphilis-exposed children is managed on a case by case basis by a pediatrician at the local maternal and child health hospital. These infants were followed up at 3, 6, 9, 12, 15, and 18 months after birth, unless congenital syphilis infection could be ruled out. During the follow-up period, the infants’ weight, height, serological test result for syphilis, and disease occurrence were measured and recorded by the pediatrician. The criteria for the exclusion of syphilis infection were met if the syphilis-exposed infant had three negative serological tests for nonspecific antibodies to syphilis at 0, 3, and 6 months of age, or if the infant had a negative TPPA test at any time during the 18-month period. If we followed a syphilis-exposed infant up to 21 months of age and were still unable to contact their caregivers and obtain any information such as growth and development indicators and infections, they were considered to have a missed visit.

Unlike the case management of syphilis-exposed children, in accordance with China’s national basic public health service standards (third edition) [18], child health physicians at primary care facilities are required to perform regular well-child checkups for all local children up to the age of 6 years.

### Data collection and statistical analysis

All data were from the China Information System for the Prevention of Mother-to-Child Transmission of Syphilis and the Jiangsu Maternal and Child Information System. The former system has been used to monitor and assess the prevalence of maternal syphilis and congenital syphilis in China. The latter is used to record the results of health check-ups for local children.

Excel 2010 was used to organize and check the data, and SPSS 20 was used for statistical analysis. The height and weight of these children conformed to normal distributions and were expressed as the mean plus or minus the standard deviation (M ± SD). A t-test was used to compare the height and weight of the children in each group with the WHO Child Growth Standards (2006 edition) [19]. Comparisons were also made with the latest growth standard for children under 7 years of age published by the National Health Commission of China (People’s Republic of China Health Industry Standard WS/T 423–2022) [20]. Multiple subgroups were analysed for differences using ANOVA, and multiple two-by-two comparisons on the basis of differences were performed using the Bonferroni correction method. *P* < 0.05 was considered to indicate statistical significance.

## Results

### Growth comparison between infants born to women with syphilis and WHO standards

By comparison with the height and weight standards promulgated by the World Health Organization in 2006, we found that syphilis-exposed infants in Changzhou were increased than the WHO standards before 18 months of age, both in height and weight, except for height at 9 months of age (Table 1). Although the length of the syphilis-exposed boys and the weight of the syphilis-exposed girls at birth did not differ from the WHO standards, they were more elevated than the standards almost at all subsequent months of growth.

**Table 1:** Growth of infants born to women with syphilis compared with WHO standards

Follow up	Indicators	Syphilis-exposed boys	WHO boys	*P*	Syphilis-exposed girls	WHO girls	*P*
at birth	Length (cm)	49.85 ± 1.36	49.88 ± 1.89	0.776	49.64 ± 1.59	49.15 ± 1.86	< 0.01
	Weight (kg)	3.44 ± 0.54	3.35 ± 0.50	0.017	3.27 ± 0.55	3.23 ± 0.47	0.267
3 months	Length (cm)	62.69 ± 2.30	61.43 ± 2.04	< 0.01	61.27 ± 2.58	59.80 ± 2.11	< 0.01
	Weight (kg)	6.96 ± 0.75	6.38 ± 0.82	< 0.01	6.40 ± 0.71	5.85 ± 0.75	< 0.01
6 months	Length (cm)	68.64 ± 2.06	67.62 ± 2.14	< 0.01	67.40 ± 2.33	65.73 ± 2.27	< 0.01
	Weight (kg)	8.56 ± 0.95	7.93 ± 0.87	< 0.01	7.97 ± 0.79	7.30 ± 0.75	< 0.01
9 months	Length (cm)	72.26 ± 2.19	71.97 ± 2.24	0.18	71.32 ± 2.23	70.14 ± 2.42	0.18
	Weight (kg)	9.63 ± 1.04	8.90 ± 1.00	< 0.01	9.02 ± 0.90	8.23 ± 1.07	< 0.01
12 months	Length (cm)	76.79 ± 2.37	75.75 ± 2.38	< 0.01	75.73 ± 2.43	74.02 ± 2.58	< 0.01
	Weight (kg)	10.53 ± 1.00	9.65 ± 1.15	< 0.01	9.97 ± 0.94	8.95 ± 1.15	< 0.01
15 months	Length (cm)	80.44 ± 2.04	79.15 ± 2.53	< 0.01	79.01 ± 2.44	77.51 ± 2.74	< 0.01
	Weight (kg)	11.50 ± 0.99	10.31 ± 1.19	< 0.01	10.69 ± 0.92	9.60 ± 1.30	< 0.01
18 months	Length (cm)	83.57 ± 3.05	82.26 ± 2.70	< 0.01	82.64 ± 2.42	80.71 ± 2.90	< 0.01
	Weight (kg)	12.12 ± 1.21	10.94 ± 1.26	< 0.01	11.47 ± 0.99	10.23 ± 1.37	< 0.01

For the continuous variables of height and weight for boys and girls, we used line graphs to present them to better focus on interpreting the mean, degree of fluctuation and outliers. From the line chart, we can see that the height of boys exposed to syphilis in the local area was higher than the World Health Organization’s standard for boys (Supplementary Fig. 1), and the height of girls showed the same (Supplementary Fig. 2).We also found that the weight of boys exposed to syphilis in the local area was greater than the standard weight (Supplementary Fig. 3), and the weight of girls showed the same (Supplementary Fig. 4).

### Growth comparison between infants born to women with syphilis and the Chinese health industry standards

Considering that these standards were published 16 years ago, they may not be suitable for evaluating the growth and development of children today. We considered it more appropriate to use the most recent Chinese Child Growth Standards. The results of this comparison are consistent with the aforementioned findings in some aspects, while differing in others. The similarity is that, at various stages from 3 months of age to 18 months of age, the weights of syphilis-exposed boys and girls were more elevated than the industry standard (*P*<0.05). However, the difference is that, the body length at 6, 12, 15, and 18 months of age for boys, as well as the body length at 6, 9, and 15 months of age for girls, were not significantly different from the industry standard(Table 2).

**Table 2:** Growth of infants born to women with syphilis compared with Chinese Health Industry Standard

Follow up	Indicators	Syphilis-exposed boys	Chinese Industry Standards boys	t-value	*P*	Syphilis-exposed girls	Chinese Industry Standards girls	t-value	*P*
at birth	Length (cm)	49.85 ± 1.36	51.2 ± 1.9	-14.799	< 0.001	49.64 ± 1.59	50.3 ± 1.9	-5.787	< 0.001
	Weight (kg)	3.44 ± 0.54	3.5 ± 0.4	-1.716	0.088	3.27 ± 0.55	3.3 ± 0.4	-0.687	0.493
3 months	Length (cm)	62.69 ± 2.30	62.2 ± 2.2	2.760	0.006	61.27 ± 2.58	60.8 ± 2.1	2.287	0.023
	Weight (kg)	6.96 ± 0.75	6.8 ± 0.8	2.878	0.004	6.40 ± 0.71	6.2 ± 0.7	3.687	< 0.001
6 months	Length (cm)	68.64 ± 2.06	68.7 ± 2.4	-0.501	0.617	67.40 ± 2.33	67.1 ± 2.3	1.727	0.086
	Weight (kg)	8.56 ± 0.95	8.4 ± 0.9	2.227	0.027	7.97 ± 0.79	7.8 ± 0.9	2.757	0.006
9 months	Length (cm)	72.26 ± 2.19	73.1 ± 2.5	-4.375	< 0.001	71.32 ± 2.23	71.5 ± 2.4	-0.984	0.327
	Weight (kg)	9.63 ± 1.04	9.4 ± 1.0	2.545	0.012	9.02 ± 0.90	8.7 ± 1.0	4.117	< 0.001
12 months	Length (cm)	76.79 ± 2.37	76.7 ± 2.6	0.353	0.725	75.73 ± 2.43	75.2 ± 2.6	2.300	0.023
	Weight (kg)	10.53 ± 1.00	10.1 ± 1.1	4.550	< 0.001	9.97 ± 0.94	9.4 ± 1.1	6.387	< 0.001
15 months	Length (cm)	80.44 ± 2.04	80.0 ± 2.7	1.815	0.075	79.01 ± 2.44	78.6 ± 2.8	1.292	0.201
	Weight (kg)	11.50 ± 0.99	10.7 ± 1.1	6.245	< 0.001	10.69 ± 0.92	10.0 ± 1.1	6.204	< 0.001
18 months	Length (cm)	83.57 ± 3.05	83.2 ± 2.9	0.706	0.483	82.64 ± 2.42	81.9 ± 2.8	2.247	0.029
	Weight (kg)	12.12 ± 1.21	11.3 ± 1.2	5.246	< 0.001	11.47 ± 0.99	10.7 ± 1.2	5.395	< 0.001

It is worth noting that both syphilis-exposed boys and girls lagged behind the Chinese industry standard in body length at birth, but both caught up by 3 months of age.

### Growth comparison between infants born to women with syphilis and the local healthy children

Being increased than the WHO standard does not tell the whole story, so we introduced local contemporaneous infants as a control group. Unlike the physical examination cycle for infants in the exposed group, infants in the control group underwent 1 physical examination at 8 months of age and no physical examination at 9 or 15 months of age. By comparing height and weight at 3, 6, 12, and 18 months of age, we found interesting points. Although syphilis-exposed children’s weight at birth did not differ from that of local infants, their weights at 12 and 18 months of age were increased than those of the local infants of the same age (Table 3). There was no difference in length from birth to 18 months of age between the two groups of boys and girls.

**Table 3:** Growth of infants born to women with syphilis compared with the local healthy infants

Indicators	Months of age	Syphilis-exposed girls (*N*_1_ = 197)	local ordinary girls (*N*_2_ = 197)	t-value	*P*-value	Syphilis-exposed boys (*N*_3_ = 222)	local ordinary boys (*N*_4_ = 222)	t-value	*P*-value
Length (cm)	at birth	49.64 ± 1.59	49.89 ± 0.82	1.954	0.052	49.85 ± 1.36	50.06 ± 0.98	1.819	0.070
	3 months	61.27 ± 2.58	61.41 ± 6.80	0.582	0.561	62.69 ± 2.30	62.72 ± 2.06	0.174	0.862
	6 months	67.40 ± 2.33	67.24 ± 2.16	-0.680	0.497	68.62 ± 2.08	68.86 ± 2.17	1.076	0.283
	8 months	—	70.48 ± 2.14	—	—	—	72.11 ± 2.18	—	—
	9 months	71.32 ± 2.23	—	—	—	72.26 ± 2.19	—	—	—
	12 months	75.73 ± 2.43	75.47 ± 2.30	-0.924	0.356	76.79 ± 2.37	76.85 ± 2.19	0.228	0.820
	15 months	79.01 ± 2.44	—	—	—	80.44 ± 2.04	—	—	—
	18 months	82.64 ± 2.42	82.22 ± 2.63	-1.003	0.317	83.57 ± 3.05	83.52 ± 2.51	-0.106	0.916
Weight (kg)	at birth	3.27 ± 0.55	3.31 ± 0.39	0.763	0.446	3.44 ± 0.54	3.39 ± 0.42	-1.083	0.279
	3 months	6.40 ± 0.71	6.50 ± 0.57	1.376	0.017	6.96 ± 0.75	7.04 ± 0.71	1.203	0.230
	6 months	7.97 ± 0.79	8.11 ± 0.79	1.720	0.086	8.56 ± 0.95	8.67 ± 0.87	1.217	0.224
	8 months	—	8.83 ± 0.89	—	—	—	9.36 ± 0.91	—	—
	9 months	9.02 ± 0.90	—	—	—	9.63 ± 1.04	—	—	—
	12 months	9.97 ± 0.94	9.75 ± 0.90	-2.009	0.045	10.53 ± 1.00	10.30 ± 0.92	-2.036	0.043
	15 months	10.69 ± 0.92	—	—	—	11.50 ± 0.99	—	—	—
	18 months	11.47 ± 0.99	11.08 ± 0.95	-2.496	0.013	12.12 ± 1.12	11.69 ± 1.03	-2.664	0.008

### Growth comparison between different groups of infants born to women with syphilis

Since there were some differences in growth in the exposed group compared to the control group, we performed further analysis. Since syphilis-infected mothers are treated with long-acting penicillin during pregnancy and their newborns receive penicillin prophylaxis after birth, and due to the lack of cooperation with treatment, this sensitive group was divided into six subgroups. They are: Group 1, where the mother received no treatment during pregnancy and the newborn was not treated after birth; Group 2, where the mother was untreated during pregnancy but the newborn received treatment after birth; Group 3, where the mother received one course of treatment during pregnancy but the newborn was untreated; Group 4, where the mother received one course of treatment during pregnancy and the newborn also received treatment; Group 5, where the mother received two courses of treatment during pregnancy but the newborn was untreated; and Group 6, where the mother received two courses of treatment during pregnancy and the newborn also received treatment. By comparing the heights and weights of the different groups, we found that there was an overall difference in the body length of the boys at 18 months of age. In a further two-by-two comparison among these six subgroups, controlling for the overall error rate, we found that the difference originated from two pairs. The group where the mother did not receive treatment during pregnancy but the newborn received treatment was shorter in length at 18 months of age than the group where the mother received one course of treatment during pregnancy and the newborn received treatment. As well, the group where the mother did not receive treatment during pregnancy but the newborn received treatment was also shorter in length at 18 months of age than the group where the mother received two courses of treatment during pregnancy and the newborn did not receive treatment (Table 4). Similarly, we also found an overall difference in the weight of the girls at 3 months of age. By performing further two-by-two comparisons, we found that the group where the mother did not receive treatment during pregnancy but the newborn received treatment weighed less at 18 months of age than the group where the mother received two courses of treatment during pregnancy and the newborn did not receive treatment (Table 5).

**Table 4:** Stratified comparison within boys born to syphilis-infected mothers

Indicators	Months of age	Syphilis-infected women not treated during pregnancy		Syphilis-infected women receive a course of treatment during pregnancy		Syphilis-infected women receive two courses of treatment during pregnancy		F-value	*P*-value
		Newborns not receiving prophylactic treatment (*N*_*1*_ = 45)	Newborns receiving prophylactic treatment (*N*_*2*_ = 21)	Newborns not receiving prophylactic treatment (*N*_*3*_ = 35)	Newborns receiving prophylactic treatment (*N*_*4*_ = 27)	Newborns not receiving prophylactic treatment (*N*_*5*_ = 49)	Newborns receiving prophylactic treatment (*N*_*6*_ = 45)		
Length (cm)	at birth	49.98 ± 1.03^*a*^	49.14 ± 1.93^*a, b,c*^	49.77 ± 1.24	49.52 ± 1.40	50.08 ± 1.50^*b*^	50.08 ± 1.11^*c*^	2.172	0.058
	3 months	62.54 ± 1.57	61.95 ± 1.86	62.58 ± 2.44	62.58 ± 2.65	62.98 ± 2.67	62.90 ± 2.37	0.673	0.644
	6 months	68.94 ± 1.85	68.24 ± 1.92	68.22 ± 2.15	68.55 ± 2.37	68.58 ± 2.43	68.91 ± 1.82	0.652	0.660
	9 months	72.52 ± 2.54	71.75 ± 1.73	72.25 ± 2.77	72.42 ± 1.90	71.93 ± 2.39	72.57 ± 1.79	0.483	0.788
	12 months	76.83 ± 2.48	76.19 ± 1.97	76.82 ± 2.65	77.35 ± 2.23	76.53 ± 3.07	76.84 ± 2.04	0.426	0.830
	15 months	80.25 ± 1.75	79.36 ± 2.16	81.00 ± 1.51	80.42 ± 2.35	82.00 ± 3.03	80.60 ± 1.45	1.490	0.209
	18 months	83.57 ± 1.90	79.25 ± 2.75^*b, d*^	82.11 ± 2.89	84.90 ± 2.96^*d*^	86.17 ± 4.07^*b*^	83.44 ± 1.90	4.275	0.003
Weight (kg)	at birth	3.57 ± 0.48^*a, e*^	3.11 ± 0.53^*a, b,c*^	3.39 ± 0.52	3.30 ± 0.72^*e*^	3.53 ± 0.56^*b*^	3.48 ± 0.42^*c*^	2.926	0.014
	3 months	7.10 ± 0.62	6.82 ± 0.72	6.71 ± 0.56	7.01 ± 0.77	6.97 ± 0.88	7.00 ± 0.82	1.056	0.387
	6 months	8.71 ± 0.81	8.53 ± 1.00	8.30 ± 0.81	8.82 ± 1.16	8.37 ± 1.04	8.66 ± 0.90	1.315	0.260
	9 months	9.77 ± 0.85	9.68 ± 1.20	9.50 ± 1.10	9.79 ± 1.18	9.29 ± 1.10	9.77 ± 0.87	0.930	0.464
	12 months	10.69 ± 0.93	10.62 ± 0.94	10.29 ± 0.89	10.89 ± 1.27	10.07 ± 1.04	10.51 ± 0.88	1.440	0.216
	15 months	11.50 ± 0.68	11.50 ± 1.31	11.08 ± 0.69	11.68 ± 1.28	11.52 ± 1.34	11.58 ± 0.63	0.364	0.871
	18 months	12.03 ± 0.82	10.98 ± 0.50	11.77 ± 1.24	12.57 ± 1.28	12.74 ± 1.33	12.11 ± 0.92	1.843	0.123

Note: *a* indicate a difference between the group 1 and group 2,*b* indicate a difference between the group 2 and group 5, *c* indicate a difference between the group 2 and group 6, *d* indicate a difference between the group 2 and group 4, *e* indicate a difference between the group 1 and group 4

**Table 5:** Stratified comparison within girls born to syphilis-infected mothers

Indicators	Months of age	Syphilis-infected women not treated during pregnancy		Syphilis-infected women receive a course of treatment during pregnancy		Syphilis-infected women receive two courses of treatment during pregnancy		F-value	*P*-value
		Newborns not receiving prophylactic treatment (*N*_*1*_ = 28)	Newborns receiving prophylactic treatment (*N*_*2*_ = 23)	Newborns not receiving prophylactic treatment (*N*_*3*_ = 20)	Newborns receiving prophylactic treatment (*N*_*4*_ = 31)	Newborns not receiving prophylactic treatment (*N*_*5*_ = 54)	Newborns receiving prophylactic treatment (*N*_*6*_ = 41)		
Length (cm)	at birth	49.39 ± 2.04	48.30 ± 2.40^*a*^	50.10 ± 1.86	49.87 ± 1.48	49.98 ± 0.46^*a*^	49.73 ± 1.21	4.799	<0.001
	3 months	60.81 ± 2.84	60.18 ± 2.75	61.89 ± 2.64	61.17 ± 2.60	61.85 ± 2.57	61.15 ± 2.12	1.645	0.151
	6 months	67.46 ± 2.57	66.27 ± 2.43	68.00 ± 2.55	66.92 ± 2.35	67.79 ± 2.02	67.71 ± 2.07	1.919	0.094
	9 months	71.59 ± 2.62	71.06 ± 2.67	72.00 ± 3.02	70.55 ± 2.11	71.35 ± 1.83	71.54 ± 1.80	0.912	0.476
	12 months	75.83 ± 3.24	75.56 ± 2.19	75.82 ± 3.22	74.58 ± 2.50	75.93 ± 2.20	76.42 ± 1.74	1.320	0.261
	15 months	79.33 ± 3.08	78.11 ± 2.09	80.00 ± 3.65	78.93 ± 3.08	79.16 ± 1.80	78.93 ± 2.37	0.391	0.853
	18 months	83.20 ± 2.39	81.86 ± 2.41	81.75 ± 4.11	82.89 ± 2.71	82.71 ± 1.94	83.10 ± 2.38	0.377	0.861
Weight (kg)	at birth	3.11 ± 0.57^*b, c*^	2.91 ± 0.67^*a, d,e, f*^	3.50 ± 0.69^*b, d*^	3.29 ± 0.59^*e*^	3.44 ± 0.57 ^*a, c*^	3.24 ± 0.58^*f*^	3.561	0.004
	3 months	6.24 ± 0.98	6.00 ± 0.80 ^*a*^	6.50 ± 0.69	6.36 ± 0.71	6.57 ± 0.54 ^*a*^	6.51 ± 0.59	2.492	0.033
	6 months	7.96 ± 1.03	7.54 ± 0.72	8.07 ± 0.77	7.88 ± 0.77	8.07 ± 0.68	8.14 ± 0.73	1.910	0.095
	9 months	9.11 ± 1.08	8.85 ± 0.61	8.89 ± 0.64	8.77 ± 0.92	9.13 ± 0.81	9.18 ± 1.11	0.828	0.532
	12 months	10.04 ± 1.43	9.92 ± 0.73	9.96 ± 0.79	9.63 ± 0.75	10.10 ± 1.09	10.07 ± 0.75	0.666	0.650
	15 months	10.75 ± 1.07	10.47 ± 1.08	10.78 ± 0.75	10.68 ± 0.99	10.53 ± 0.98	11.00 ± 0.70	0.549	0.739
	18 months	11.66 ± 1.07	11.84 ± 0.84	11.08 ± 0.99	11.26 ± 0.92	11.33 ± 0.93	11.64 ± 1.28	0.534	0.750

Note: *a* indicate a difference between the group 2 and group 5, *b* indicate a difference between the group 1 and group 3, *c* indicate a difference between the group 1 and group 5, *d* indicate a difference between the group 2 and group 3, *e* indicate a difference between the group 2 and group 4, *f* indicate a difference between the group 2 and group 6

## Discussion

In our study, it made sense that the infants born to syphilis-infected mothers were more elevated than the WHO reference standard for growth and development across the board. On the one hand, the WHO reference standards were published 16 years ago and may be less applicable for evaluating the growth and development of infants today [19]. Between 1985 and 2019, China had the world’s greatest increase in average height for 19-year-old males and the world’s third greatest increase for 19-year-old females [21]. On the other hand, Jiangsu’s GDP per capita, regional development and livelihood index all rank first among China’s provinces, making it one of China’s highest comprehensive development provinces, and Jiangsu is already at the “upper-middle” developed country level. The combination of good material conditions and good health concepts will not cause children to lag behind in terms of growth and development.

Our comparison with the latest Chinese industry standards shows that both boys and girls still lead in weight across the board, but there is no difference in length for more than half of them. There was no difference in height between the syphilis-exposed but uninfected group and the unexposed control group, but the syphilis-exposed group had heavier weights at 12 and 18 months of age than the control group. Even if the differences were statistically significant, the differences were all within 1 standard deviation, which could mean that the differences were due to sampling error or measurement error. If these errors were eliminated by increasing the sample size or by good quality control, or if the results were not different, the differences that currently exist may not be clinically significant; however, they deserve our attention.

This result is approximately the same as the results of a study conducted in Zhejiang, a neighbouring province, during the same period [22]. Additionally, in Zhejiang Province, China, a retrospective analysis showed that after 6 and 12 months of age, the height and body mass of newborns delivered by single-infected mothers and those delivered by multiinfected mothers were significantly lower than those of newborns delivered by healthy mothers [10], which is completely different from our results. This is probably because the infections in that study were mainly HBV infections, although syphilis infections were present. The number of cases in the infected group was only 86 and that in the healthy group was only 50, which may cause problems due to an insufficient sample size and poor sample representation. In contrast, a follow-up study conducted on children with congenital syphilis in Beijing showed that there was a tendency for children with congenital syphilis treated with regular syphilis expulsion to be physically stunted, with the most pronounced growth impairment observed in length development [8]. Since approximately 2 cases of congenital syphilis occur in our region each year, our next step is to conduct a multicentre study to compare the growth and development indicators of children with congenital syphilis with those of various paediatric populations.

One study revealed that infants born to mothers who receive formal anti-syphilis treatment during pregnancy had greater developmental indicators than infants born to mothers who received informal treatment, but the difference was not statistically significant [9]. Our findings were essentially the same, with both boys and girls differing in height or weight at only 1 time point, although their mothers received different levels of intervention. Syphilis spirochetes can cross the placenta leading to mother-to-child transmission of syphilis, but it appears that the incidence of congenital syphilis can be greatly reduced as long as the mother or newborn is treated with penicillin. Another study revealed no marked differences in the incidence of low birth weight (LBW) between childbearing women with and without syphilis [22]. Therefore, we hypothesize that as long as congenital syphilis does not progress, it will not affect the growth and development of the child. The half-life of long-acting penicillin is approximately 7 days, and it is metabolized in the body in approximately one month, and does not cause any long run term effects [23]. Gregorio Serra’s team reported a case of growth retardation after birth in a premature infant with congenital syphilis, but the report emphasized the possibility that this condition may be the result of an uncommon clinical presentation of CS [24]. A study conducted last year in Hunan Province, China, showed that maternal syphilis infection during pregnancy was associated with a 2.60-fold (95% CI 1.83–3.69) increased risk of low birth weight and a 1.91-fold (95% CI 1.35–2.69) increased risk of preterm birth. Similarly, a review published in recent years cites syphilis infection during pregnancy as a possible determinant of fetal growth restriction [13]. However, in our study, such differences did not seem to be reflected in the subsequent growth and development of the children.

Although we tried to minimize or homogenize the known influences as much as possible in the grouping process, there are still some potential limitations that may interfere with the stability of the results. For example, there may be differences in feeding practices within 18 months of age, and the use of long-acting penicillin as a treatment regimen may have an impact on the microflora of breast milk; breastfed children may have imbalances in specific intestinal flora and metabolite abnormalities, which could progressively affect the growth and development of syphilis-exposed infants and toddlers [25]. Regarding preventive intervention strategies, in addition to the antibiotics analyzed in this study, future considerations could include the impact of vaccination methods, as suggested by case reports related to rotavirus [26]. These questions deserve further investigation by our team. In the future, timely early screening of pregnant women and appropriate interventions during pregnancy and delivery will reduce the likelihood of vertical transmission from mother to child and improve the development of newborns born to infected mothers.

## Conclusion

We found little difference in the growth and development curves of infants who were exposed to but not infected with syphilis and infants who were not exposed to syphilis. We also found that the length and weight of syphilis-exposed infants were more elevated than the World Health Organization’s standards published in 2006 across the board, and there was little difference compared to China’s industry standards published in 2023. Whether or not pregnant women with syphilis infection received treatment during pregnancy and whether or not their newborns received prophylactic treatment after birth appeared to differ only in individual subgroups from further subgroup analyses. In conclusion, receiving a single long-acting penicillin injection as early as possible after birth does not have a negative impact on infant growth and may improve the effectiveness of mother–infant transmission prevention. The differences in a few subgroups of infants of different ages may be due to the insufficient sample size, and we will improve this in the future. Neonatologists and pediatricians have a key role to play in preventing adverse growth and developmental outcomes in such at-risk children, including the potentially beneficial clinical, social, and economic effects of early intervention [27]. 

## Electronic supplementary material

Below is the link to the electronic supplementary material.

Supplementary Material 1

Supplementary Material 2: **Fig. 1.** Height trend chart of syphilis-exposed boys of different months compared with WHO standards. **Fig. 2.** Height trend chart of syphilis-exposed girls of different months compared with WHO standards. **Fig. 3.** Weight trend chart of syphilis-exposed boys of different months with WHO standards. **Fig. 4.** Weight trend chart of syphilis-exposed girls of different months with WHO standards

Supplementary Material 3

## Data Availability

The datasets GENERATED for this study can be found in the JIANGUOYUN. https://www.jianguoyun.com/c/sd/16b6d3b/5dc7e57997b32902#from=https%3 A%2 F%2Fwww.jianguoyun.com%2Fc%2Fsd%2F16b6d3b%2F5dc7e57997b32902.
