# Supplementary material for: The effect of benzylpenicillin prohylaxis after birth on length and weight of syphilis-exposed infants in eastern China
Source: Ital J Pediatr. 2024 Sep 27;50:199. doi: 10.1186/s13052-024-01779-7 (PMC11438286; doi:10.1186/s13052-024-01779-7)
Supplement: Supplementary file 2 — Supplementary Material 2: Fig. 1. Height trend chart of syphilis-exposed boys of different months compared with WHO standards. Fig. 2. Height trend chart of syphilis-exposed girls of different months compared with WHO standards. Fig. 3. Weight trend chart of syphilis-exposed boys of different months with WHO standards. Fig. 4. Weight trend chart of syphilis-exposed girls of different months with WHO standards [file 13052_2024_1779_MOESM2_ESM.docx]

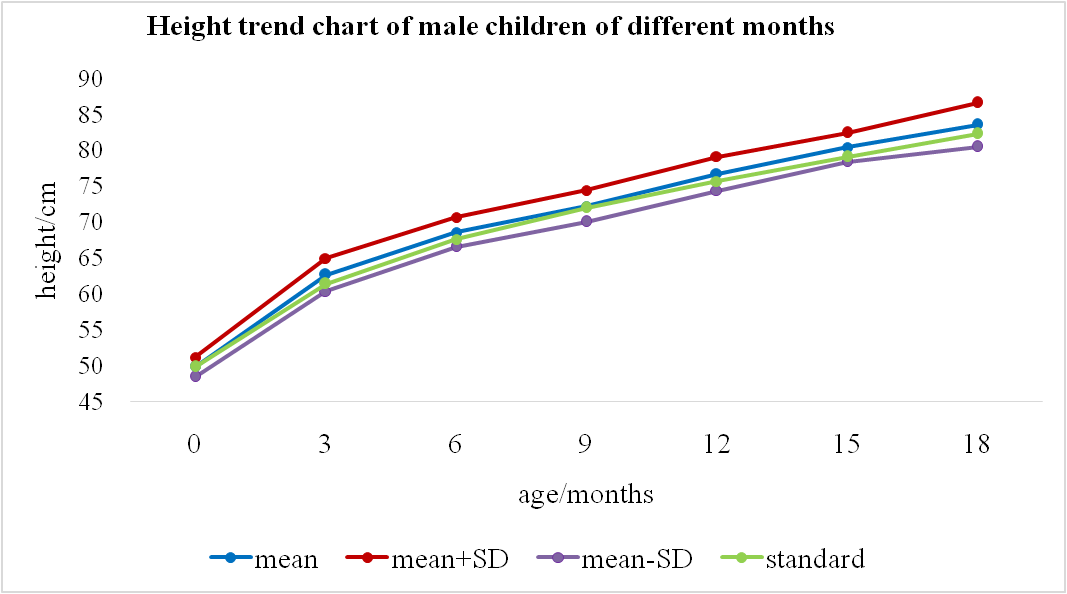


**Figure 1** Height trend chart of syphilis-exposed boys of different months compared with WHO standards


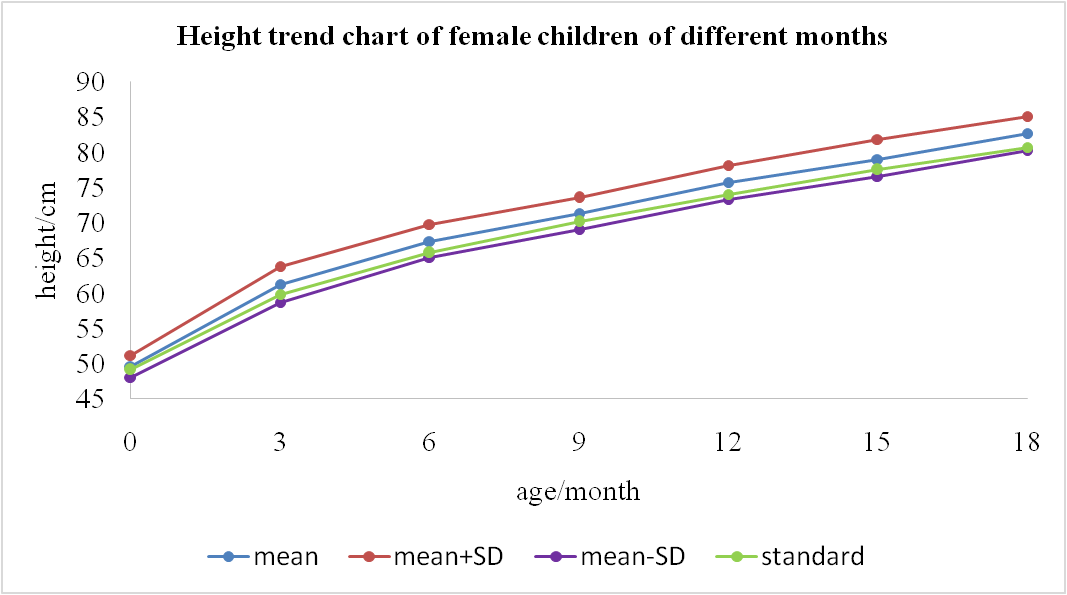


**Figure 2** Height trend chart of syphilis-exposed girls of different months compared with WHO standards


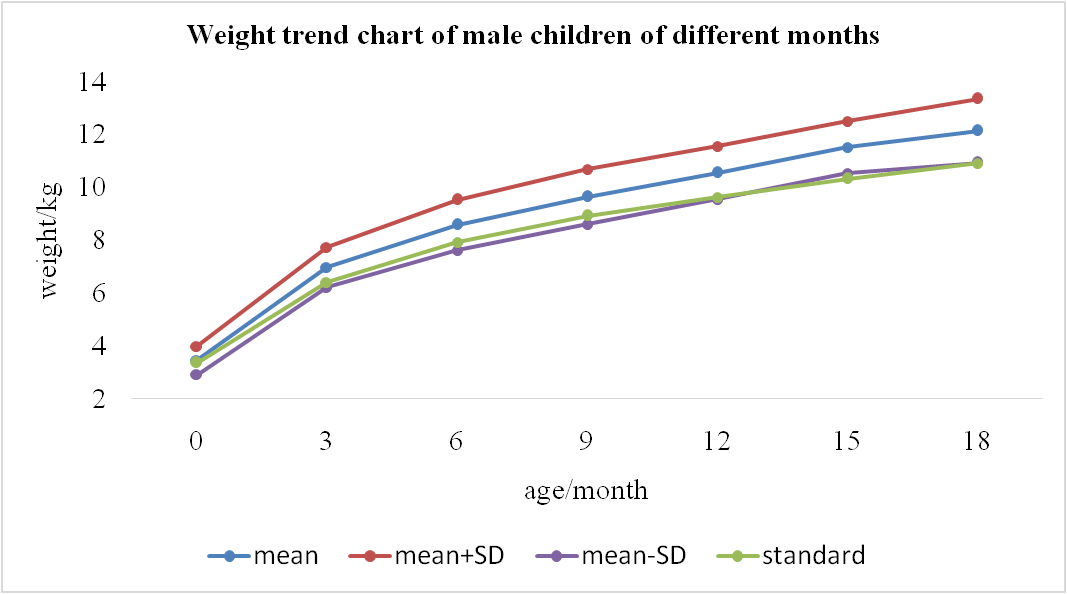


**Figure 3** Weight trend chart of syphilis-exposed boys of different months compared with WHO standards


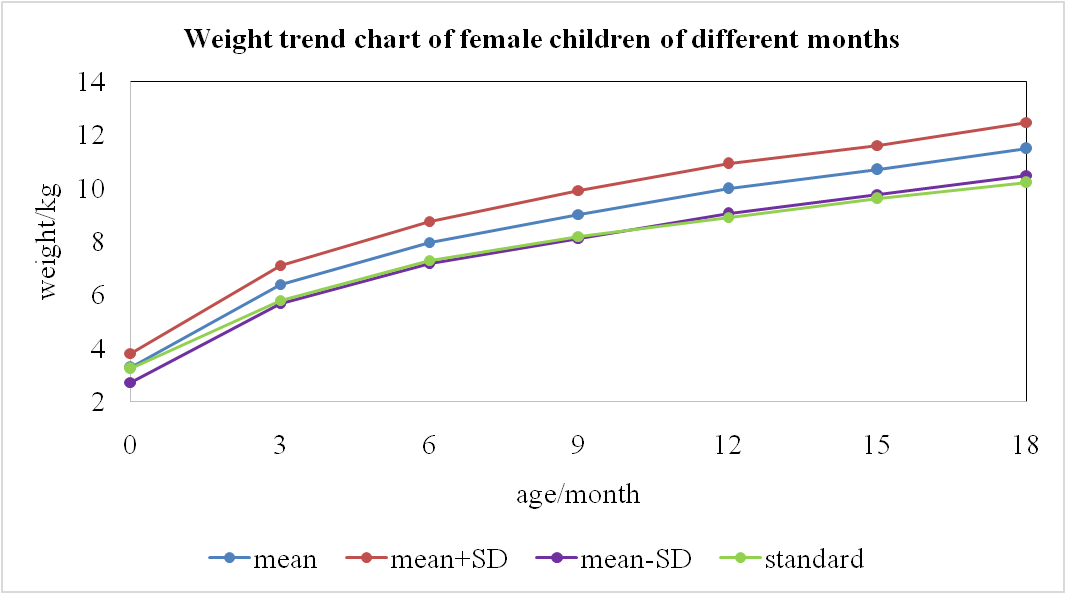


**Figure 4** Weight trend chart of syphilis-exposed girls of different months compared with WHO standards
