# Supplementary material for: The effect of benzylpenicillin prohylaxis after birth on length and weight of syphilis-exposed infants in eastern China
Source: Ital J Pediatr. 2024 Sep 27;50:199. doi: 10.1186/s13052-024-01779-7 (PMC11438286; doi:10.1186/s13052-024-01779-7)
Supplement: Supplementary file 1 — Supplementary Material 1 [file 13052_2024_1779_MOESM1_ESM.doc]

Dear Editor:

We would like to submit the enclosed manuscript entitled “The effect of preventive treatment after birth on morphological indicators of syphilis-exposed infants in eastern China”, which we wish to be considered for publication in “Italian Journal of Pediatrics”.

Although numerous studies have demonstrated the importance of maternal-infant interruption of syphilis, it is unclear whether the growth and development of syphilis-exposed children is actually affected. In our study, the development of children exposed to syphilis was compared with World Health Organization standards. We also matched 1:1 children born to syphilis-free mothers during the same period，and compared with them. It is worth pointing out, grouping mothers and newborns by the criterion of whether they received treatment or not, and then performing further subgroup analyses.

Our conclusion is that the growth and developmental trajectory of children born to syphilis-infected mothers without congenital syphilis is virtually indistinguishable from that of the local general population. Our study will further provide favourable evidence for syphilis parturients and their newborns to receive standardized treatment.

We confirm that neither the manuscript nor any parts of its content are currently under consideration or published in another journal.

We previously submitted to Clinical Epidemiology, and the manuscript was rejected after peer review. We have made revisions based on the comments of the three reviewers to further improve the quality of the article.

All authors have approved the manuscript and agree with its submission to Italian Journal of Pediatrics.

I hope this paper is suitable for “Italian Journal of Pediatrics”.

We deeply appreciate your consideration of our manuscript, and we look forward to receiving comments from the reviewers. If you have any queries, please don’t hesitate to contact me at the address below.

Sincerely,
*Chaorong Bian,MD
Changzhou Children’s Hospital Affiliated to Nantong University*
*No.958, Zhongwu Road, Changzhou, Jiangsu Province, China*
Email address: *babysbreathabc@163.com*
Telephone: *(+86)* *15961434931*
