# Supplementary material for: The effect of benzylpenicillin prohylaxis after birth on length and weight of syphilis-exposed infants in eastern China
Source: Ital J Pediatr. 2024 Sep 27;50:199. doi: 10.1186/s13052-024-01779-7 (PMC11438286; doi:10.1186/s13052-024-01779-7)
Supplement: Supplementary file 3 — Supplementary Material 3 [file 13052_2024_1779_MOESM3_ESM.docx]

#Reviewer reports:

Congratulations to the authors who greatly improved their manuscript in clarity and overall quality, overcoming the critical issues raised.

Only a few and small changes are requested before publication.

A: Thank you once again for taking the time to carefully review our revised article. We will continue to improve the quality of the article based on the following issues you raised.

Q1: In the title, the "preventive treatment" seems aspecific, and could be improved clearly changing it with "The effect of benzylpenicillin prohylaxis after birth on length and weight of syphilis-exposed infants in eastern China".

A1: Thank you for your comments. In the title, replacing "preventive treatment" with "benzylpenicillin prohylaxis" can indeed help readers understand and interpret the article more accurately and easily. We have made revisions in this regard.

Q2: Main manuscript-line 47, about the preventive intervention strategies, also vaccination approach (for virus like HBV or rotavirus) and antifungal prohylaxis to neonates should be included and cited by authors, besides that with antibiotics like the one analzyed in the present study (and useful against bacterial infections). These measures are useful as well to improve growth and global outcomes and to prevent children from the infection-related detrimental effects and complications. In regard to this point add the following report:

Acute pancreatitis in children and rotavirus infection. Description of a case and minireview. S. Giordano, G. Serra, P. Dones, M. Di Gangi, M.C. Failla, C. Iaria, F. Ricciardi, L.M. Pernice, D. Pantaleo, A. Cascio. New Microbiologica. 2013 Jan;36(1):97-101.

A2: Thank you for your comments. Our team discussed and considered placing this sentence at the end of the discussion section, as follows:

"Although we tried to minimize or homogenize the known influences as much as possible in the grouping process, there are still some potential limitations that may interfere with the stability of the results. For example, there may be differences in feeding practices within 18 months of age, and the use of long-acting penicillin as a treatment regimen may have an impact on the microflora of breast milk; breastfed children may have imbalances in specific intestinal flora and metabolite abnormalities, which could progressively affect the growth and development of syphilis-exposed infants and toddlers.^25^ Regarding preventive intervention strategies, in addition to the antibiotics analyzed in this study, future considerations could include the impact of vaccination methods, as suggested by case reports related to rotavirus.^26^ These questions deserve further investigation by our team. In the future, timely early screening of pregnant women and appropriate interventions during pregnancy and delivery will reduce the likelihood of vertical transmission from mother to child and improve the development of newborns born to infected mothers."

Q3: Growth comparison-line 168, the results of this comparison are both similar to and different from those above? please clarify as it seems contradictory.

A3: Thank you for your comments. As you mentioned, this sentence is somewhat inadequately expressed here. Therefore, we made some changes and added a few connecting words, as follows:

"The results of this comparison are consistent with the aforementioned findings in some aspects, while differing in others. The similarity is that, The results of this comparison are both similar to and different from those above. At at various stages from 3 months of age to 18 months of age, the weights of syphilis-exposed boys and girls were more elevated than the industry standard(P＜0.05). However, the difference is that, the body length at 6, 12, 15, and 18 months of age for boys, as well as the body length at 6, 9, and 15 months of age for girls, were not significantly different from the industry standard(Table 2). "

Q4: Growth comparison-line 188, I would remove "development" as there is no mention at all about the evaluation of this aspect

A4: Thank you for your comments. Based on your suggestion, we have removed "and development".

Q5: Growth comparison-line 192, it would be better for major clarity to list the six different subgroups outlined, as made in table 4.

A5: Thank you for your comments. Based on your suggestion, we have provided detailed information on the subgroup classifications, as follows:

"They are: Group 1, where the mother received no treatment during pregnancy and the newborn was not treated after birth; Group 2, where the mother was untreated during pregnancy but the newborn received treatment after birth; Group 3, where the mother received one course of treatment during pregnancy but the newborn was untreated; Group 4, where the mother received one course of treatment during pregnancy and the newborn also received treatment; Group 5, where the mother received two courses of treatment during pregnancy but the newborn was untreated; and Group 6, where the mother received two courses of treatment during pregnancy and the newborn also received treatment. "
